# Meningoencephalitis in a background of inherited chromosomic integration of HHV-6 and CMV infection in an immunocompetent adult, which one is the culprit?

**DOI:** 10.1016/j.idcr.2025.e02219

**Published:** 2025-04-03

**Authors:** Anne-Laure Lajoye, Doina Ciocanu, Gilles Duverlie, Patrick Berquin

**Affiliations:** a Service de Neurologie Pédiatrique, CHU Amiens-Picardie, France; b Service de Neurologie, CH Lisieux, France; c Laboratoire de Virologie, CHU Amiens-Picardie, France

**Keywords:** Meningitis, Encephalitis, Cytomegalovirus, Human herpesvirus 6, Immunocompetent, Inherited chromosomal integration

## Abstract

HHV-6 meningoencephalitis has been reported in immunocompromised individuals but is very uncommon in immunocompetent individuals. However, HHV-6 is the second most frequently detected virus in multiplex PCR tests. As HHV-6 DNA integrates in the telomeric region of host chromosomes after primary infection and can be passed onto offspring, 1 % of the population carries an inherited HHV-6 genome (iciHHV-6). This makes it difficult to interpret a positive multiplex PCR test for HHV-6. Here, we describe a 39-year-old female patient with an unremarkable medical history and who was hospitalized for meningoencephalitis. The brain imaging findings were normal. The positive multiplex PCR test for HHV-6 was confirmed by qualitative and quantitative HHV-6 PCR tests, the viral load was higher in blood than in CSF. The presence of circulating anti-CMV IgM and IgG in a serologic test and the absence of other pathogens argued in favor of a primary CMV infection. However CMV PCR was negative. The chromosomal integration of HHV-6 was subsequently confirmed by the results of a hair bulb analysis. Our primary hypothesis was CMV meningoencephalitis in a context of inherited chromosomic integration of HHV-6 without be able to confirm the possible role of HHV-6 (reactivation or bystander) in this situation. A commercially available assay able to certify HHV-6 replication in a context of iciHHV-6 would have been useful to conclude.

## Introduction

### Human herpesvirus 6 (HHV-6)

HHV-6 is an enveloped, double-stranded linear DNA opportunistic virus that is closely related to cytomegalovirus (CMV). HHV-6 is the collective name for 2 unique species of herpesviruses (HHV-6A and HHV-6B), and while both have distinct cell tropisms, both are also neurotropic and lymphotropic. The HHV-6B is responsible for the vast majority of clinical disease.

Primary infection usually occurs before the age of 3 years and results in exanthema subitem. By the start of adulthood, more than 90 % of individuals have been in contact with HHV-6A or B. The virus persists in a latent state, with limited expression of viral genes and no production of infectious viral particles. Reactivation is frequent and is typically asymptomatic in immunocompetent adults but clinically severe in immunocompromised people, such as those taking immunosuppressive drugs or having undergone hematopoietic stem cell transplantation [1], [4]. In immunocompetent patients, symptomatic reactivations of HHV-6 can be triggered by severe sepsis, co-infections with CMV [3] or herpes simplex virus (HSV), or the use of certain drugs (e.g. amoxicillin) [11], [2].

Like other viruses (HIV, lentiviruses) HHV-6 integrates into the host genome. However, it is the only one that establishes latency through the telomeric region of chromosomes (ciHHV-6) and that can be vertically passed onto offspring when integration occurs in germline cells, known as inherited chromosomal integration of HHV-6 (iciHHV-6). In the case of iciHHV-6, at least one copy of the HHV-6 genome is present in every cell with a 1:1 ratio of HHV-6 DNA and human cell DNA. This induces a positive viral load in all cellular samples tested: cerebrospinal fluid (CSF HHV-6 PCR) and serum (viral load), due to cellular apoptosis with release of cellular DNA. Approximately 1 % of the population is thought to carry iciHHV-6 [15].

### Multiplex PCR

The BioFire® Filmarray® is the most commonly used diagnostic assay in cases of meningoencephalitis and can detect 14 different pathogens within an hour. HHV-6 is the second most frequently detected micro-organism (after enterovirus) in multiplex PCR assays of CSF [8]. When the PCR assay is positive for HHV-6 (i.e. the presence of viral genomic DNA in the CSF), one must then try to distinguish between primary infection, latent chromosomal integration, and viral reactivation (i.e. active replication, whether symptomatic or not). At present, a technique for determining the nature of the HHV-6 transcripts is not available in routine clinical practice. Quantitative PCR assays of whole blood (yielding the viral load) or CSF cannot differentiate between genomic integration or active viral replication, although a viral load > 5.5 log_10_ copies/mL whole blood is much higher than that found in patients with an active primary infection and thus is strongly suggestive of iciHHV-6 [15], [17].

Definitive confirmation of chromosomal integration can be achieved by (i) quantitative PCR testing of a nail or hair bulb sample (because only patients with iciHHV-6 have viral DNA in these tissues) or (ii) droplet digital PCR on whole blood, in order to determine the viral DNA/host DNA ratio (1:1 for individuals with iciHHV-6) [15].

Although HHV-6 meningoencephalitis is very rare in immunocompetent individual and is mainly described in hematopoietic stem cell recipients [6], the causal role of HHV-6 in meningitis in an immunocompetent patient must be considered carefully by reviewing all the clinical and laboratory data.

Here, we describe a case of meningoencephalitis in an immunocompetent young woman with concomitant CMV seroconversion and only HHV-6 in the CSF.

## Case report

A 39-year-old woman came to our emergency department following the onset of aphasia earlier that day, after an unusual, week-long, progressively worsening headache. She was not feverish and did not report having been in contact with people with an ongoing infection. A clinical examination confirmed the presence of revealed persistent, isolated, fluent, expressive aphasia with neologisms and jargonaphasia. The results of brain MRI (stroke protocol) and a contrast-enhanced CT scan of the brain and the supra-aortic trunks were unremarkable.

The patient was transferred to the neurology department. After lumbar puncture, the CSF contained 283 white blood cells (WBCs)/mm^3^ (lymphocytes: 87 %; 1 hematocyte). The CSF protein level was 0.75 g/L, and the CSF glucose level was normal. No microbes were found by direct microscopic examination of the CSF.

While waiting for the multiplex PCR results, we initiated empiric antiviral treatment with acyclovir and antiepileptic treatment with levetiracetam. On day 2 of the hospital stay, the multiplex PCR assay was positive for HHV-6 only; this finding was confirmed by a quantitative HHV-6 PCR assay (18,876 HHV-6 DNA copies/CSF mL, i.e., 4.28 log_10_ HHV-6 DNA copies/mL).

On day 6, the results of a second brain MRI with gadolinium contrast agent were normal. A second lumbar puncture was performed on D7. Analysis of the CSF sample revealed pleocytosis (420 WBCs; lymphocytes: 98 %), a CSF protein level of 1.95 g/L, and a normal CSF glucose level. The multiplex PCR assay did not detect any other bacteria or viruses, and the qualitative PCR assay for HHV-6 was still positive. Furthermore, a quantitative PCR assay of a blood sample gave a viral load of 14,200,000 IU/mL (7.15 log_10_ IU/mL). A serologic test for HHV-6 was also positive, with an IgG index of 20 but no IgM. Neither an inflammatory syndrome nor signs of a possible immunodeficiency were present: the serum protein electrophoresis profile and diabetes markers were normal, and the serologic tests for HIV, SARS-CoV-2, HSV1 and HSV2 were negative. Only the serologic test result for CMV was suggestive of a recent infection (anti-CMV IgG: 350 IU/mL; anti-CMV IgM: 1.63 IU/mL), although qualitative CMV PCR assays on CSF and blood samples were both negative.

The aphasia initially resolved completely upon treatment with acyclovir but reocurred for 24 h on D3. A persist headache prompted us to reinitiate treatment with ganciclovir IV 5 mg/kg on D8, together with amitriptyline.

On day 3 (during the course of treatment with levetiracetam), the electroencephalogram showed some signs of degradation in the left frontotemporal region against a normal background recording. A electroencephalogram recorded on day 5 was normal, and the levetiracetam was discontinued.

A third lumbar puncture was performed on D11. The HHV-6 viral load in CSF was 337,924 HHV-6 DNA copies/CSF mL in quantitative PCR assay, i.e. 5,53 log_10_/CSF mL. Compared with the first lumbar puncture, a higher cell count of 420 WBC/mm^3^ was founded in the same time and CSF protein level was 1.95 g/L. CMV PCR assay in CSF remained negative.

As the viral load of HHV-6 in the blood was greater than 10^6^ IU/mL, we used a quantitative PCR assay to screen a hair bulb sample for ciHHV-6. Ganciclovir was discontinued after 10 days of treatment, and amitriptyline was continued until the headaches disappeared. The outcome at 1 month was favorable, with no sequelae.

One month later, a positive PCR test for the hair bulb sample (1 228 500 HHV-6 DNA copies/mL, 6 log_10_ copies/mL or 4 136 363 DNA copies/10^6^ cells) confirmed the genomic integration.

## Discussion

In the present case, we detected iciHHV-6 with a CMV infection (i.e., blood seroconversion without active CMV replication in the CSF). Positivity of HHV-6 PCR in this context of inherited chromosomic integration did not enable us to distinguish between latent HHV-6 integration and active replication. It has to be noted that HHV-6 PCR was higher in blood than in CSF, which could sustain the hypothesis of HHV-6 latency. Assays on CSF samples carried out on days 2 and 7 were negative for both CMV serology and CMV DNA but may have been conducted too early to detect the virus. After reconsidering all the biological results we had and the major efficacy of gangiclovir, our main hypothesis was CMV meningoencephalitis in a context of iciHHV-6, even if it remains hard to tell whether iciHHV-6 or CMV was the real culprit.

Since the introduction of multiplex PCR assays, the number of reported cases of HHV-6 meningitis or encephalitis has increased significantly [9]. Most HHV-6-positive CSF samples indirectly reflect chromosomal integration and are considered to be clinically irrelevant [14]. In Berzero et al.’s 10-year retrospective study, none of the immunocompetent individuals developed HHV-6 encephalitis after the age of 3 years or upon viral reactivation. Although a few case reports of HHV-6 meningitis or encephalitis following primary infection or reactivation in immunocompetent adults have been published, this condition is still very rare [2], [10], [12], [18].

There are currently no routinely available laboratory tests for determining whether an individual with iciHHV-6 also has an active HHV-6 infection. Amplification of HHV-6 gives no information about the activity of iciHHV-6, which is transcriptionally silent almost all of the time. To provide evidence of iciHHV-6 reactivation, assays providing HHV-6 lytic protein expression or transcriptome sequencing (small non-coding RNAs analysis or microRNA analysis) would be needed [14], [16]. Unfortunately, no such assays are commercially available.

HHV-6 reactivation in our case could be further debated as IgM were absent. Their presence would have supported the hypothesis of HHV-6 infection although can be missing. In the article by [5], only half of the HHV-6 reactivations were detected by both an increase in IgG antibodies and presence of IgM antibodies. 40 % of the remaining patients had only a rise in IgG without IgM.

**Fig. 1:**
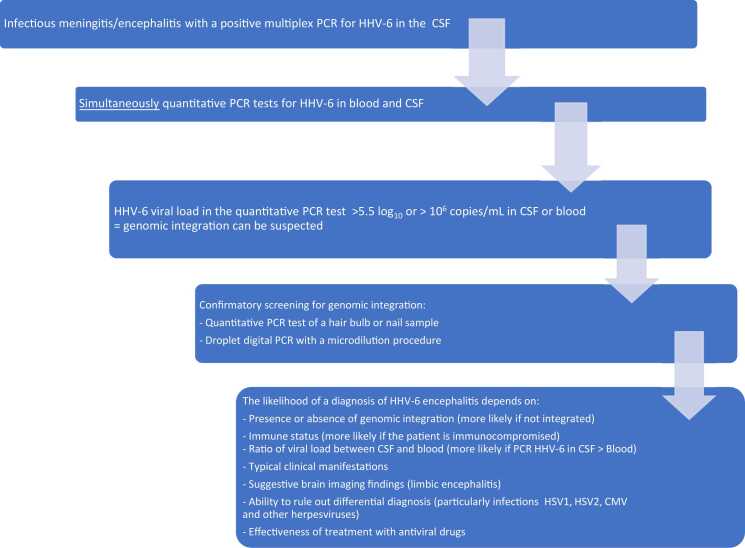
The diagnostic work-up prompted by a positive multiplex PCR for HHV-6 in a case of meningitis or encephalitis.

In a case of meningoencephalitis, the possible causal role of HHV-6 and the appropriate adapted antiviral treatment must be considered by taking several factors into account (Fig. 1).

We lack a specific, validated treatment for HHV-6 meningoencephalitis in immunocompetent adults, given the rarity of this condition [7], [10], [15]. By analogy with the antivirals used to treat CMV infections, foscarnet and ganciclovir can be administered intravenously and valganciclovir can be administered orally [15].

CMV meningoencephalitis is rare in immunocompetent adults but nevertheless appears to be more common than HHV-6 encephalitis in the same context [13]. Mozafarybazargany et al. described a 48-year-old immunocompetent patient who presented with fatal CMV encephalitis; no micro-organisms were found in CSF samples collected by lumbar puncture on admission and on D5. Only the CSF sample collected after three weeks contained detectable CMV. Thus, screening the CSF might not be useful in the context of an early CMV infection. In our patient, the results of quantitative PCR test of blood and CSF samples did not confirm a causal role for CMV in the meningoencephalitis. However, the CMV infection might have triggered replication of HHV-6. Unfortunately, we did not ask for CMV IgG and IgM analysis in CSF. If positive, a higher CMV IgM titer in CSF than in blood would have sustained the hypothesis of CMV causal infection.

## Consent

The patient provided her written consent to the publication of this article, a copy of which will be stored in her medical records.

## Conclusion

We have described a possible case of HHV-6 meningoencephalitis following reactivation of an inherited chromosomally integrated virus concomitantly with a primary CMV infection. In immunocompetent adults, HHV-6 meningoencephalitis is very rare. When considering HHV-6’s possible causal role in meningoencephalitis, a positive multiplex PCR test for HHV-6 always raises the question of whether HHV-6 has integrated into the chromosome; in an immunocompetent individual, iciHHV-6 is the most frequent reason for the detection of HHV-6 DNA in the CSF. A routinely available laboratory test for determining the origin of viral transcripts and thus the cause of active viral replication is now urgently needed. RT-PCR tests must be more widely available, more sensitive and more specific.

## CRediT authorship contribution statement

**Anne-Laure Lajoye:** Writing – review & editing, Writing – original draft. **Doina Ciocanu:** Supervision. **Gilles Duverlie:** Validation. **Berquin Patrick:** Writing – review & editing, Supervision.

## Consent

Written informed consent was obtained from the patient for publication of this case report and accompanying images. A copy of the written consent is available for review by the Editor-in-Chief of this journal on request.

## Ethical approval

Does not apply

## Declaration of Interest

None.
